# Author Correction: The chromosomal organization of horizontal gene transfer in bacteria

**DOI:** 10.1038/s41467-020-15091-5

**Published:** 2020-02-26

**Authors:** Pedro H. Oliveira, Marie Touchon, Jean Cury, Eduardo P. C. Rocha

**Affiliations:** 1 Microbial Evolutionary Genomics, Institut Pasteur, 25–28 rue du Docteur Roux, Paris, 75015 France; 2 CNRS, UMR3525, 25–28 rue du Docteur Roux, Paris, 75015 France

Correction to: *Nature Communications* 10.1038/s41467-017-00808-w, published online 10 October 2017.

The original version of this Article contained an error in Eq. 2. This equation incorrectly included a 2 in the denominator, and incorrectly read: $\beta _{{\mathrm{SIM}}} = \frac{{\mathop {\sum}\nolimits_{i < j} {{\mathrm{min}}\left( {b_{ij}, \, b_{ji}} \right)} }}{{2 \cdot \left( {\mathop {\sum}\nolimits_i {S_i \, - \, S_T} } \right) \, + \, \mathop {\sum}\nolimits_{i < j} {{\mathrm{min}}\left( {b_{ij}, \, b_{ji}} \right)} }}$.

The correct form of Eq. 2 is: $\beta _{{\mathrm{SIM}}} = \frac{{\mathop {\sum}\nolimits_{i < j} {{\mathrm{min}}\left( {b_{ij},\, b_{ji}} \right)} }}{{\left( {\mathop {\sum}\nolimits_i {S_i \, - \, S_T} } \right) \, + \, \mathop {\sum}\nolimits_{i < j} {{\mathrm{min}}\left( {b_{ij}, \, b_{ji}} \right)} }}$

All the calculations of BSIM are correct and were made following the correct form of Equation 2.

This has been corrected in the PDF and HTML versions of the Article.

