# Supplementary material for: The chromosomal organization of horizontal gene transfer in bacteria
Source: Nat Commun. 2017 Oct 10;8:841. doi: 10.1038/s41467-017-00808-w (PMC5635113; doi:10.1038/s41467-017-00808-w)
Supplement: Supplementary file 1 — Supplementary Information [file 41467_2017_808_MOESM1_ESM.pdf]

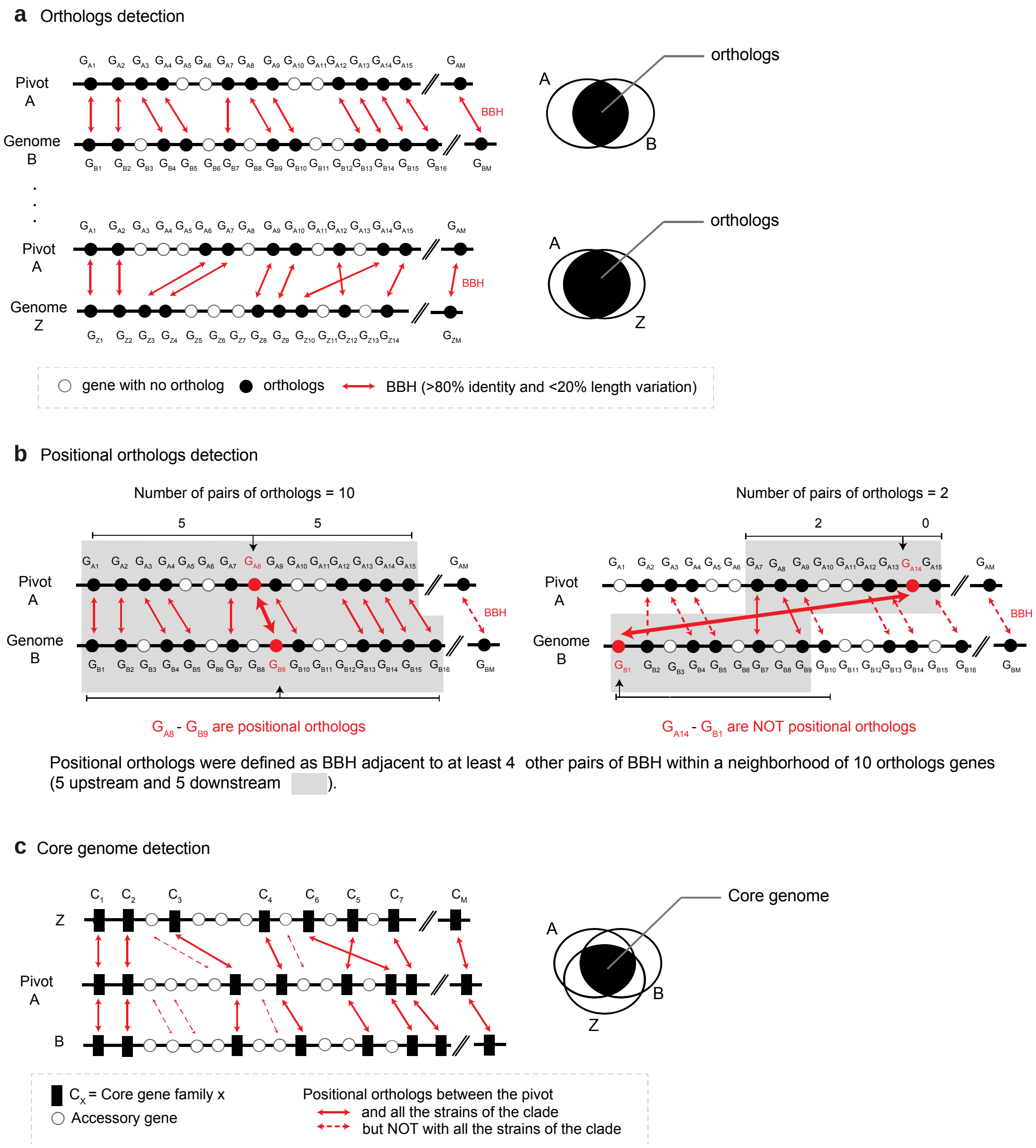

Supplementary Fig. 1. Core genome identification was done in three steps. (a) A preliminary list of orthologs was identified as reciprocal best hits (BBH) using end-gap free global alignment, between the proteome of a reference genome A (pivot) and each of the other strain's proteomes of the clade (*e.g.,* B to Z). (b) Positional orthologs were defined as BBH adjacent to at least 4 other pairs of BBH within a neighborhood of 10 ortholog genes (5 upstream and 5 downstream = gray window). Examples of positional orthologs (left) and non-positional orthologs (right). (c) The core genome of each clade was defined as the intersection of pairwise lists of positional orthologs. C_X corresponds to the core gene family x, where x is the position along the pivot core-genome (here the genome A).

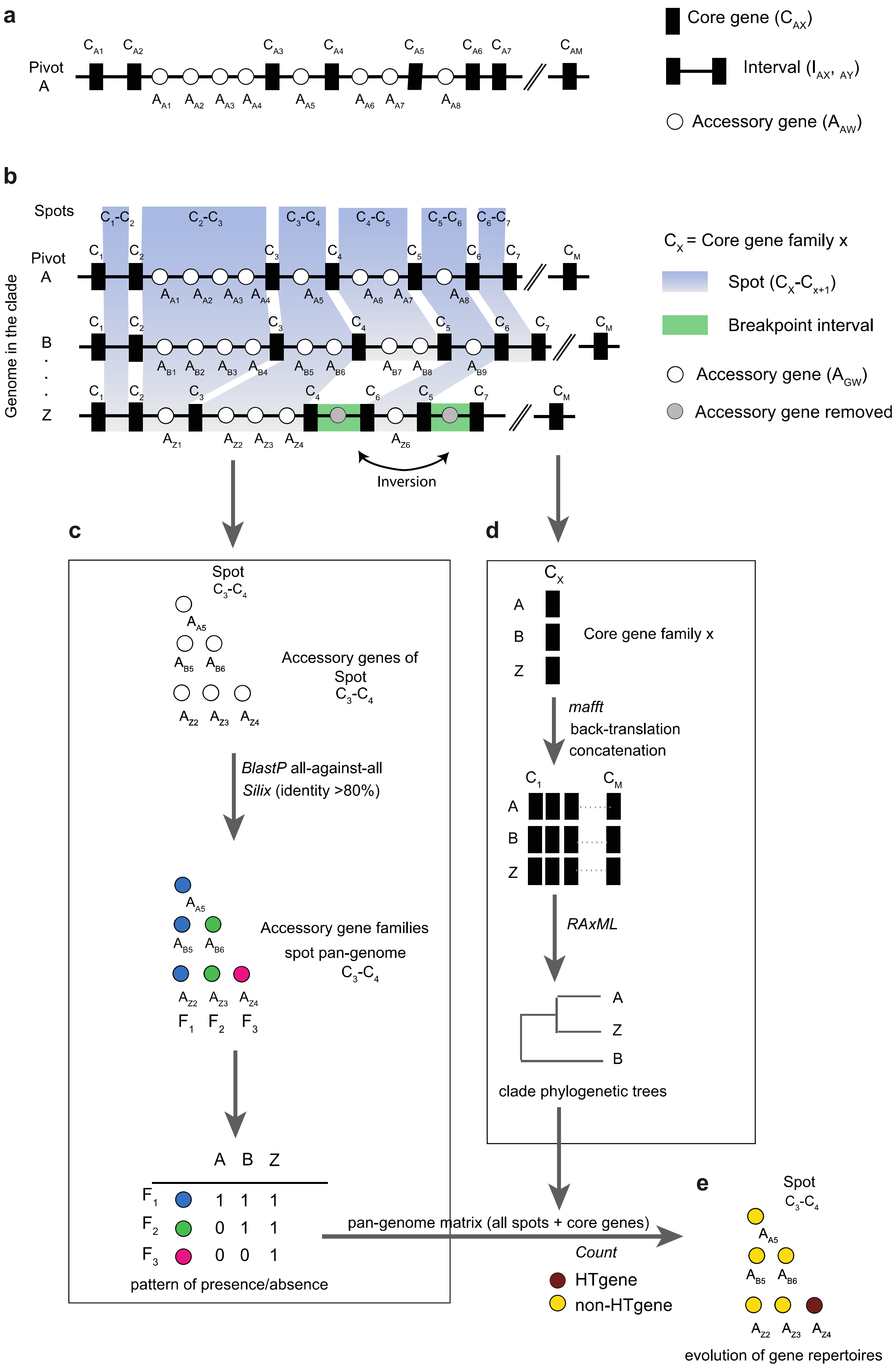
a
Pivot A
Core gene (C_{AX})
Interval ($I_{AX, AY}$)
Accessory gene (A_{AW})
b
Spots
Genome in the clade
Pivot A
B
Z
Inversion
C_X = Core gene family x
Spot (C_X-C_{x+1})
Breakpoint interval
Accessory gene (A_{GW})
Accessory gene removed
c
Spot C_3-C_4
Accessory genes of Spot C_3-C_4
BlastP all-against-all
Silix (identity >80%)
Accessory gene families spot pan-genome C_3-C_4
F_1 F_2 F_3
A B Z
F_1 1 1 1
F_2 0 1 1
F_3 0 0 1
pattern of presence/absence
d
C_X
Core gene family x
mafft
back-translation
concatenation
RAxML
clade phylogenetic trees
pan-genome matrix (all spots + core genes)
Count
HTgene
non-HTgene
e
Spot C_3-C_4
evolution of gene repertoires

Supplementary Fig. 2. (a) Scheme depicting key concepts used in this study. Intervals (I_{AX}, $_{AY}$) were defined by consecutive core genes in the list of core genes sorted by position in chromosome A. (b) Intervals flanked by the same core gene families (C_X, C_Y) as those from pivot genome A were defined as *syntenic intervals* (*i.e.*, the members of the core gene families X and Y were also contiguous in the pivot). The intervals that do not satisfy this constraint were classified as *breakpoint intervals* (green-shaded regions) and excluded from our analysis. For every interval in the pivot genome, we defined a *spot* as the set of intervals flanked by members of the same core gene families (blue-shaded regions). (c) For each *spot* we clustered in the same gene family (F) accessory genes sharing at least 80% identity and with coverage relatively to the smallest protein higher than 80%. Thus, the *spot pan-genome* is the set of gene families present in the *spot* (*e.g.* in the spot C_3-C_4). We then computed the spot pan-genome matrix that corresponds to the pattern of presence/absence of each gene family present in the spot in each genome of the clade. (d) We made core genome trees for each clade using a concatenate of the multiple alignments of the core genes. Each species tree was computed with RAxML under the GTR model and a gamma correction (GAMMA) for variable evolutionary rates. We performed 100 bootstrap experiments on the concatenated alignments of each clade to assess the robustness of the topology of the tree. (e) We used the spots' pan-genomes matrices, the phylogenetic tree, and the phylogenetic birth-and-death model of Count, to evaluate the most likely scenario for the evolution of a given gene family on the clade's tree.

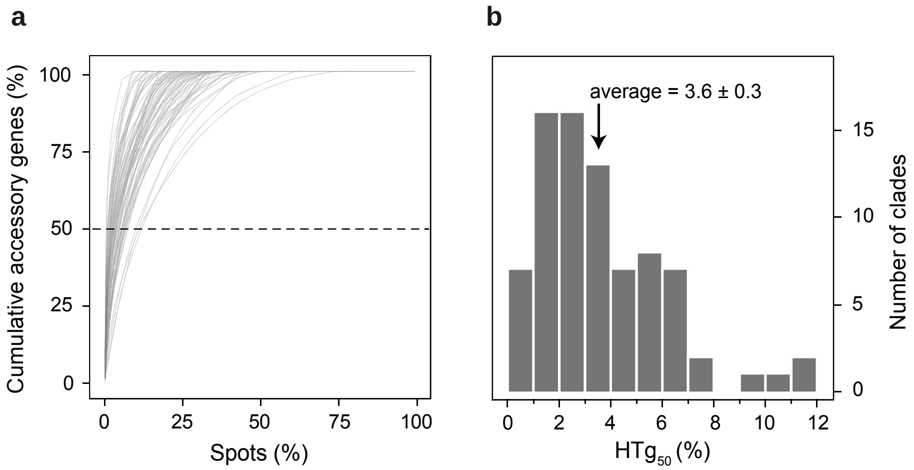

Supplementary Fig. 3. (a) Cumulative distribution of the total accessory genes (%) in spots for the 80 bacterial clades. (b) Histogram of the minimum number of the largest spots needed to attain 50% of the accessory genome (HTg_{50} index) in each clade. Here, the average HTg_{50} was 3.6% (± 0.3; standard deviation). Thus, 50% of the accessory genes were on average, in the 3.6% larger spots and were slightly less clustered than HTgenes ($P < 10^{-4}$; Mann–Whitney–Wilcoxon test).

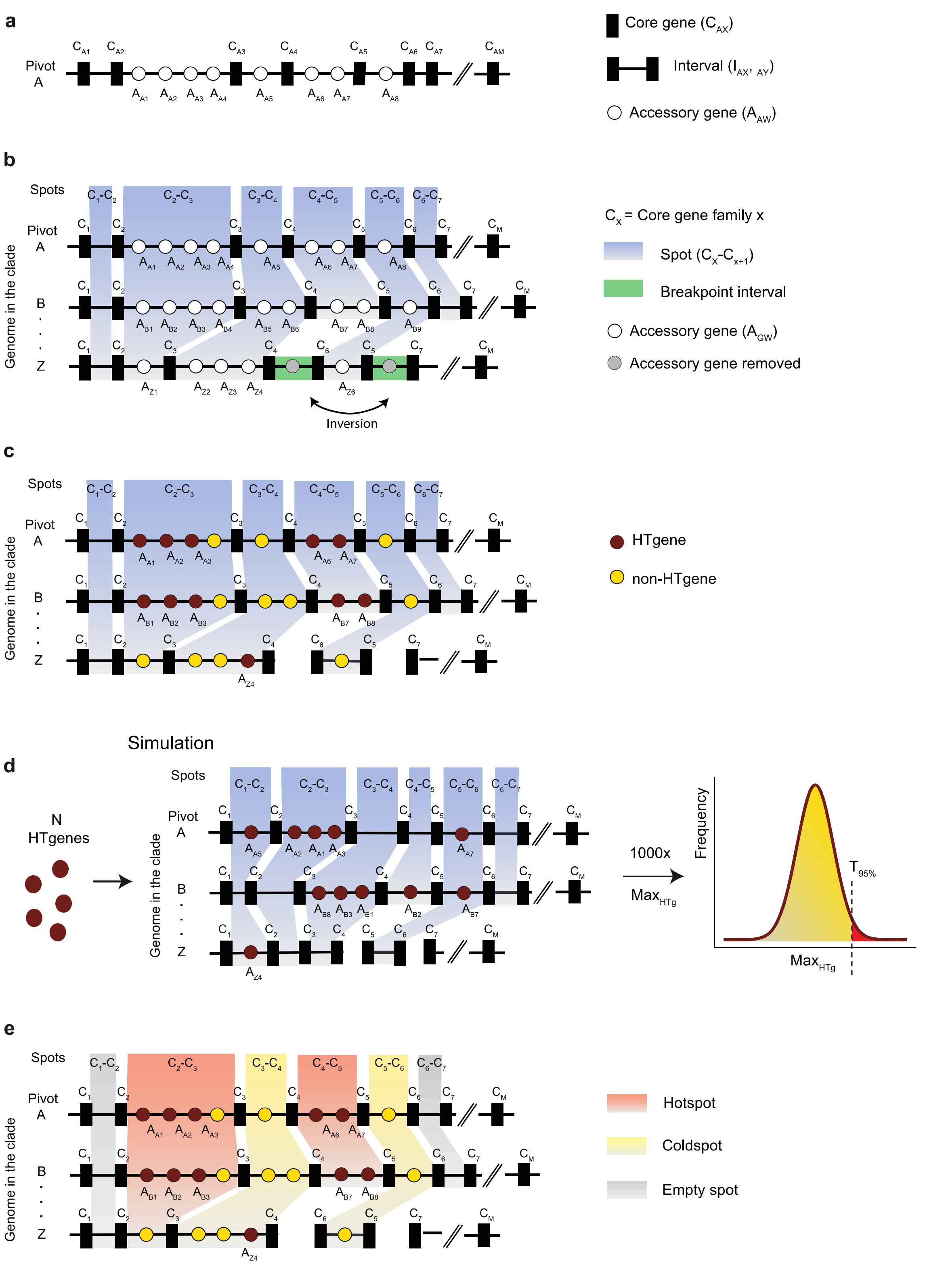

Supplementary Fig. 4. (a) Scheme depicting key concepts used in this study. Intervals (I_{AX}, $_{AY}$) were defined by consecutive core genes in the list of core genes sorted by position in the chromosome A. (b) Intervals flanked by the same core gene families (C_X, C_Y) as the pivot

genome A were defined as *syntenic intervals* (*i.e.*, the members of the core gene families X and Y were also contiguous in the pivot). The intervals that do not satisfy this constraint were classed as *breakpoint intervals* (green-shaded regions) and excluded from our analysis. For every interval in the pivot genome, we defined *spot* as the set of intervals flanked by members of the same core gene families (blue-shaded regions). (c) Using information provided by Count on the dynamics of gene repertoires of each clade, we distinguished HTgenes (red circles) from the other accessory genes (yellow circles). (d) In order to identify hotspots, we used a uniform distribution to draw random numbers and distribute 1,000 times the *N* HTgenes throughout the *M* spots of each clade. This distribution was performed either considering that all accessory genes are singletons, or considering that 1/3 are singletons and the remaining 2/3 are clustered in operons of 3 genes each (Supplementary Fig 5). The maximum number of integrated genes at a given spot was recorded for each simulation ($Max_{HTg,i}$). Finally, for each species, we took the 95th percentile ($T_{95\%}$) of the distribution of $Max_{HTg,i}$. The results of $T_{95\%}$ are shown in Supplementary Dataset 1. Because the simulation allowing both singletons and operons is more accurate from a biological standpoint, we have chosen its thresholds for the classification of spots as hotspots. (e) Scheme representing spots as either hotspots (number of HTgenes $>T_{95\%}$, red-shaded regions), empty (genes absent, gray-shaded regions), or coldspots (the remaining, yellow-shaded regions).

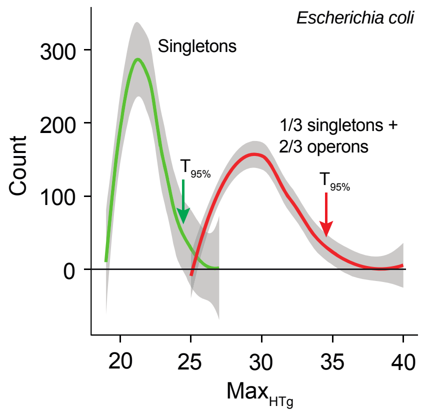

Supplementary Fig. 5. Example of how $T_{95\%}$ is computed for *E. coli*. The graph shows the distribution of the maximum numbers of HTgenes (Max_{HTg}) in one spot when simulating uniform repartition of those genes 1,000 times. This distribution was computed either considering that all genes are singletons (in green), or considering that 1/3 are singletons and the remaining 2/3 are clustered in operons of 3 genes each (in red).

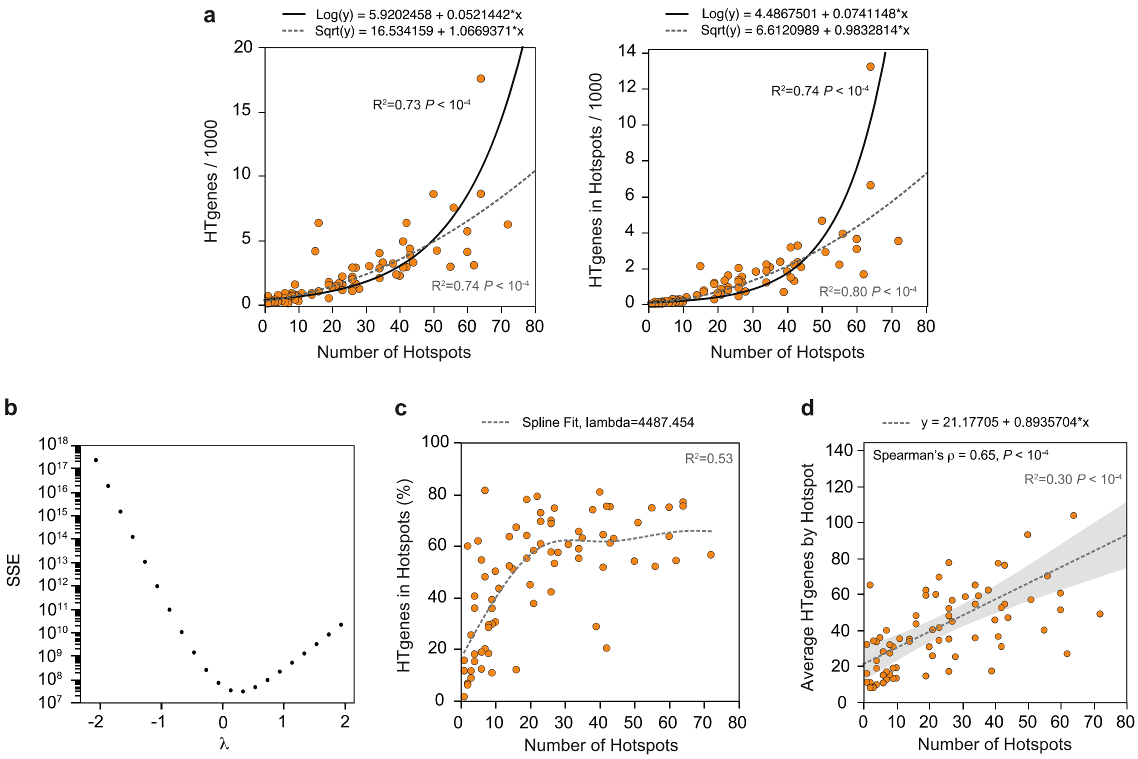

Supplementary Fig. 6. (a) Association between the number of hotspots and the total number of HTgenes in each clade (left) and the number of HTgenes within hotspots (right). In both, log (full) and square-root (dashed line) regressions and the corresponding R^2 were reported. (b) Box-Cox analysis of the relation between total HTgenes and the number of hotspots. The minimal value of SSE (sum of squared errors) is observed for values of λ in the Box-Cox transformation intermediate between a log and a square-root transformation (lambda = 1 would be linear, *i.e.*, the best association would be a straight line). (c) Proportion of HTgenes in hotspots in function of the number of hotspots in each clade. The spline fit (dashed line) and corresponding R^2 are indicated. (d) Association between the average number of HTgenes by hotspot and the number of hotspots in clades. The linear regression (dashed line), and the corresponding R^2 and Spearman's ρ are indicated.

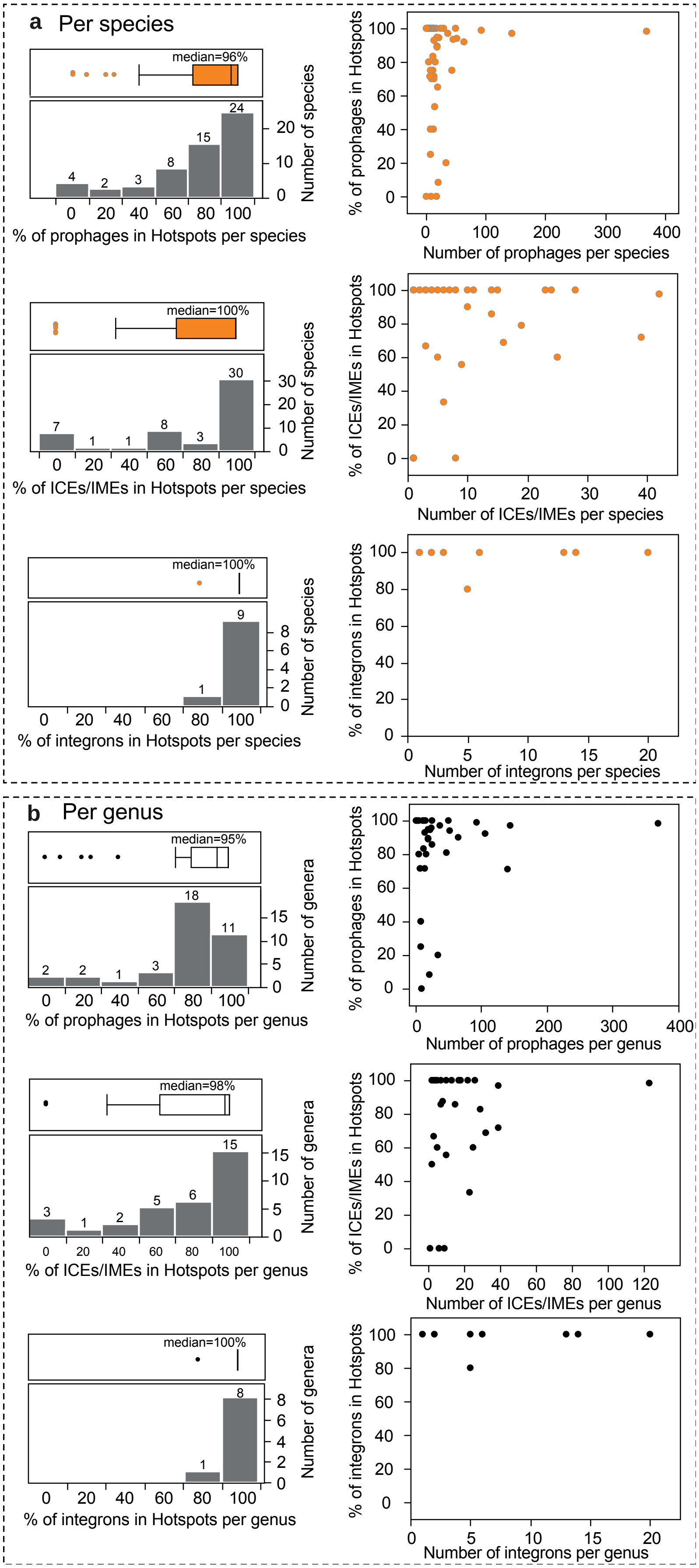
a
Per species
median=96%
24
15
8
4
2
3
Number of species
% of prophages in Hotspots per species
% of prophages in Hotspots
Number of prophages per species
median=100%
30
7
1
1
8
3
% of ICEs/IMEs in Hotspots per species
% of ICEs/IMEs in Hotspots
Number of ICEs/IMEs per species
median=100%
9
1
% of integrons in Hotspots per species
% of integrons in Hotspots
Number of integrons per species
b
Per genus
median=95%
18
11
2
2
1
3
Number of genera
% of prophages in Hotspots per genus
Number of prophages per genus
median=98%
15
3
1
2
5
6
% of ICEs/IMEs in Hotspots per genus
Number of ICEs/IMEs per genus
median=100%
8
1
% of integrons in Hotspots per genus
Number of integrons per genus

Supplementary Fig. 7. Distribution of the percentage of each type of MGE (prophages, ICEs/IMEs, and integrons) in hotspots per species (a) and per genus (b). (Left) The median of the distribution is indicated in the boxplots. The number of species on each bin is indicated on top of each bar of the histograms. (Right) Percentage of each type of MGE in hotspots according to the number of each type of elements detected per species (a) or genus (b). The results are also shown in Supplementary Dataset 3b-c.

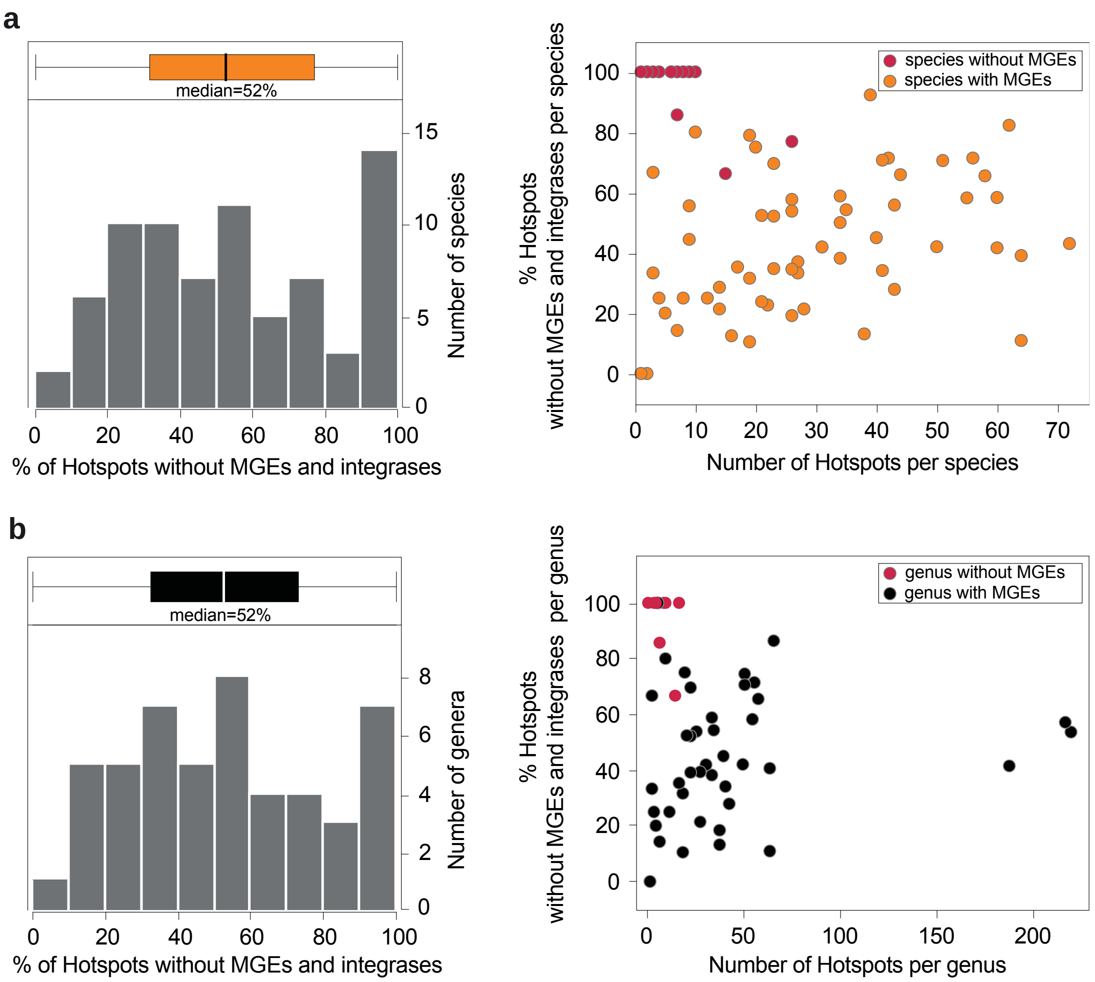

Supplementary Fig. 8. (Left) Distribution of the percentage of hotspots without MGEs (*i.e.* prophages, ICE/IMEs and Integrons) and integrases per species (a) and per genus (b). The median of the distribution is indicated in the boxplots. (Right) Percentage of hotspots without MGEs and integrases according to the total number of hotspots. In our dataset, 14 species (belonging to 8 genera) with hotspots are devoid of MGEs in their genomes (red dots). The results are shown in Supplementary Dataset 3d-e.

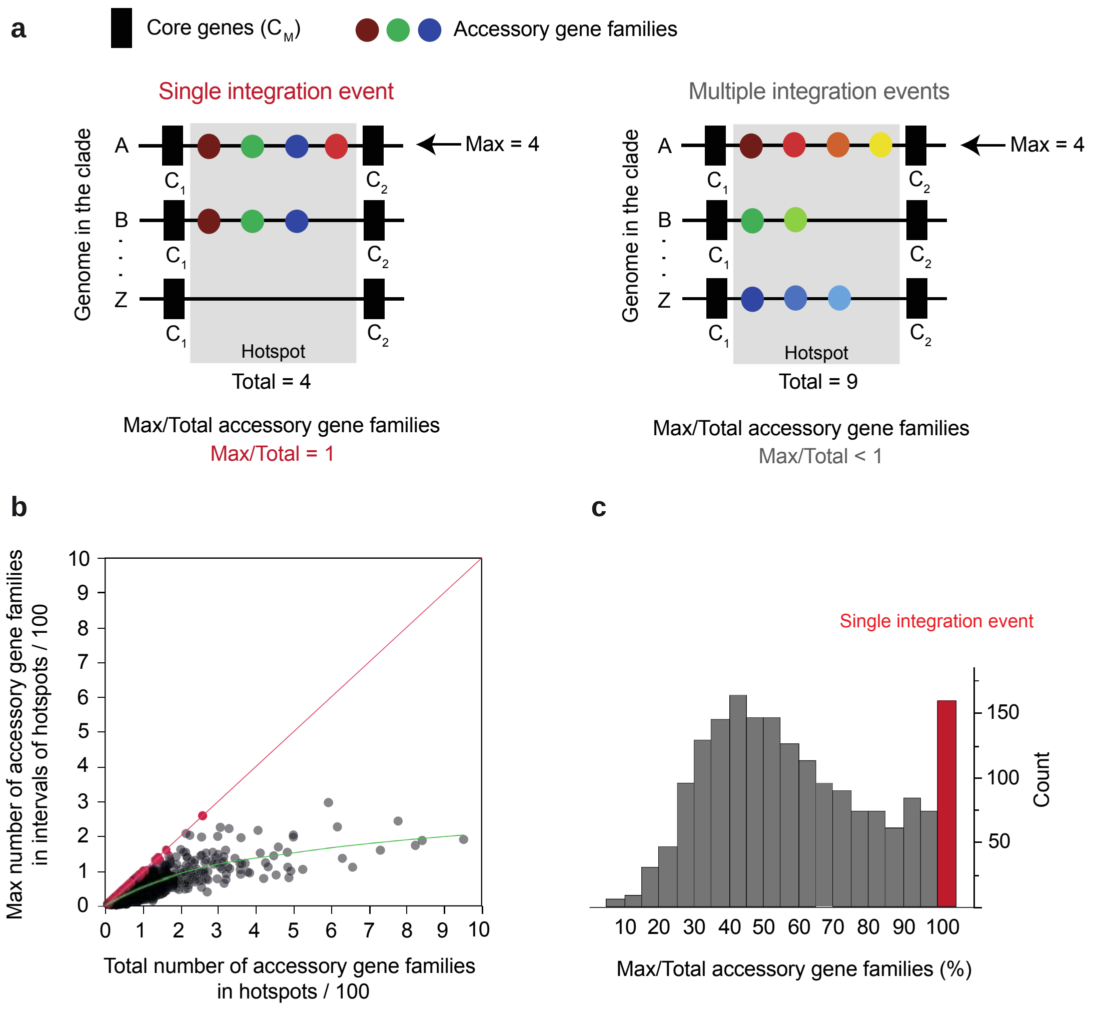

Supplementary Fig. 9. (a) Examples of a single integration and multiple integration events in a hotspot. In the former, at least one genome should have all the diversity of the hotspot pan-genome. Hence, the maximal number of accessory gene families (Max) observed in at least one genome should be equal to the hotspot pan-genome (Total). It should not be the case for multiple integration events (Max/Total<1). (b) The maximal number of accessory gene families (Max) in function of the total number of accessory gene families (Total) in each hotspot. The red dots correspond to single integration event (Max/Total=1). (c) Distribution of the ratio (Max/Total %), the red bar corresponds to single integration event.

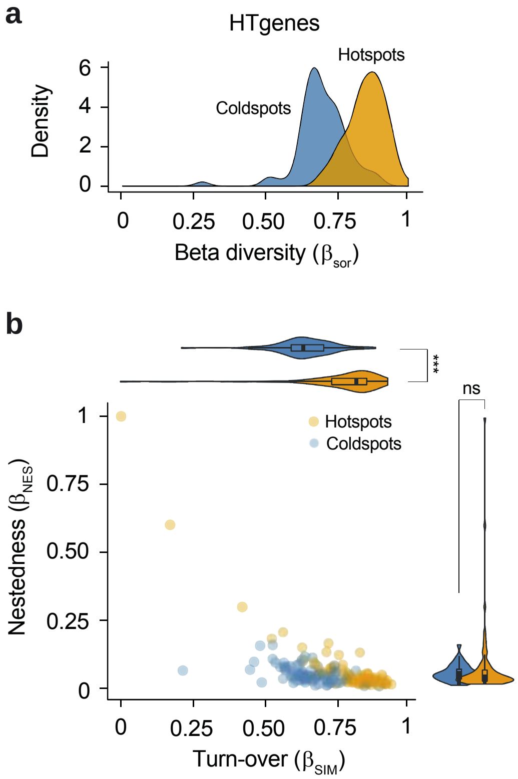

Supplementary Fig. 10. Genetic diversity of the HTgenes present in hotspots and coldspots. (a) Distributions of β diversity (β_{SOR}) in hotspots and coldspots. (b) Partition of β_{SOR} in its components of nestedness (β_{NES}) and turnover (β_{SIM}) for hotspots and coldspots. (*** $P < 10^{-3}$; Mann–Whitney–Wilcoxon test; ns: not significant).

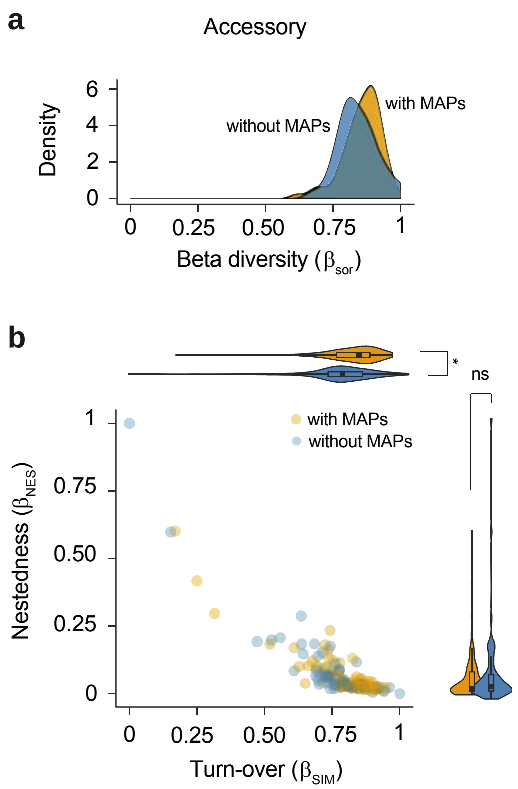

Supplementary Fig. 11. Genetic diversity of the accessory genes present in hotspots with or without MAPs. (a) Distributions of β diversity (β_{SOR}). (b) Partition of β_{SOR} in its components of nestedness (β_{NES}) and turnover (β_{SIM}) (* P < 0.05; Mann–Whitney–Wilcoxon test; ns: not significant).
