# Supplementary material for: The chromosomal organization of horizontal gene transfer in bacteria
Source: Nat Commun. 2017 Oct 10;8:841. doi: 10.1038/s41467-017-00808-w (PMC5635113; doi:10.1038/s41467-017-00808-w)
Supplement: Supplementary file 3 — Description of Additional Supplementary Files [file 41467_2017_808_MOESM3_ESM.pdf]

Description of Additional Supplementary Files

File name: Supplementary Data 1
Description: List of the 80 bacterial species analyzed in this study.

File name: Supplementary Data 2
Description: 16S rRNA phylogenetic tree used to build Fig. 2a and phylogenetic trees for each of the 80 bacterial species studied

File name: Supplementary Data 3
Description: Number of hotspots with or without mobile genetic elements or proteins-associated to mobility computed per species and per genus.

File name: Supplementary Data 4
Description: The origin and terminus of replication predicted by Ori-Finder in the pivot genome of each clade.

File name: Supplementary Data 5
Description: List of median values of nucleotide diversity, homologous recombination, and SH test P value in core genes flanking hotspots and non-hotspots computed per species and per genus.
